# Superspreading in early transmissions of COVID-19 in Indonesia

**DOI:** 10.1038/s41598-020-79352-5

**Published:** 2020-12-28

**Authors:** Agus Hasan, Hadi Susanto, Muhammad Firmansyah Kasim, Nuning Nuraini, Bony Lestari, Dessy Triany, Widyastuti Widyastuti

**Affiliations:** 1 Mærsk McKinney Møller Institute, University of Southern Denmark, Odense, Denmark; 2 Department of Mathematics, Khalifa University, Abu Dhabi, United Arab Emirates; 3 Department of Mathematical Sciences, University of Essex, Colchester, UK; 4 Department of Physics, University of Oxford, Oxford, UK; 5 Department of Mathematics, Institut Teknologi Bandung, Kota Bandung, Indonesia; 6 Department of Epidemiology and Biostatistics, Universitas Padjadjaran, Sumedang, Indonesia; 7 Public Health Office of Batam City, Batam City, Indonesia; 8 Public Health Office of Jakarta Province, Jakarta, Indonesia

**Keywords:** Diseases, Health care

## Abstract

This paper presents a study of early epidemiological assessment of COVID-19 transmission dynamics in Indonesia. The aim is to quantify heterogeneity in the numbers of secondary infections. To this end, we estimate the basic reproduction number $\mathscr {R}_0$ and the overdispersion parameter $\mathscr {K}$ at two regions in Indonesia: Jakarta–Depok and Batam. The method to estimate $\mathscr {R}_0$ is based on a sequential Bayesian method, while the parameter $\mathscr {K}$ is estimated by fitting the secondary case data with a negative binomial distribution. Based on the first 1288 confirmed cases collected from both regions, we find a high degree of individual-level variation in the transmission. The basic reproduction number $\mathscr {R}_0$ is estimated at 6.79 and 2.47, while the overdispersion parameter $\mathscr {K}$ of a negative-binomial distribution is estimated at 0.06 and 0.2 for Jakarta–Depok and Batam, respectively. This suggests that superspreading events played a key role in the early stage of the outbreak, i.e., a small number of infected individuals are responsible for large numbers of COVID-19 transmission. This finding can be used to determine effective public measures, such as rapid isolation and identification, which are critical since delay of diagnosis is the most common cause of superspreading events.

## Introduction

The first two confirmed cases of COVID-19 in Indonesia was announced in March 2, 2020 by the president himself. Jakarta and its buffer zones including Depok have become the epicenter of the outbreak. In its early transmission, about 50% of cases were from the cities. The virus quickly spread to all 34 provinces, with more than 450,000 confirmed cases and 15,000 deaths as of November 12, 2020. As a country with the highest death toll in South East Asia and one with the lowest global testing rate, the first government effort to control the disease was by establishing a COVID-19 response acceleration task force led by the head of the National Disaster Management Agency. The government further introduced Large-Scale Social Restrictions, which included measures such as closing public places, restricting public transport, and limiting travel to and from restricted regions. On May 19, the first milestone of 10,000 PCR tests per day was reached. In June 2020, WHO announced that only Jakarta meets the minimum requirement of 1 test per 1000 population per week for a reliable positivity rate calculation.

As the Indonesian government struggles to mitigate the COVID-19 epidemic in the country, it becomes critical to understand its infection spreads. Here, we present a first quantitative analysis of early transmission dynamics of the disease and the evidence of superspreading events in Indonesia based on contact tracing of secondary cases. The aim is to quantify heterogeneity in numbers of secondary infections. Infection clusters are difficult to predict and thus difficult to prevent. Nevertheless, understanding their environmental and behavioral drivers have been recently recognized to be extremely important to inform strategies for prevention and control^1,2^ as the reproduction number is no longer enough^3^. Previous studies in China and Hong Kong show identifying and interrupting superspreading events are critical importance of preventing the spread of the infection^4,5^.

## Methods

With regard to transmission, whenever we find a new COVID-19 case, we labelled them as index cases. Aiming to contain the transmission, we then perform contact tracing to identify persons who have close contact with the index cases and send them for testing when necessary. In our study, any close contacts of the index case with positive COVID-19 test based on the Indonesian Guideline for COVID-19 Control were defined as secondary cases. Thereafter, we can calculate the average number of secondary cases generated from a set of index cases. In our analysis of Jakarta, we combine data from Depok that is a main buffer city of the capital. Outside the epicentre, we present data from Batam that has received thousand test kits and protective equipment from Singapore as a comparison. There were 218 cases from 4092 PCR tests, as of June 26.

**Figure 1:**
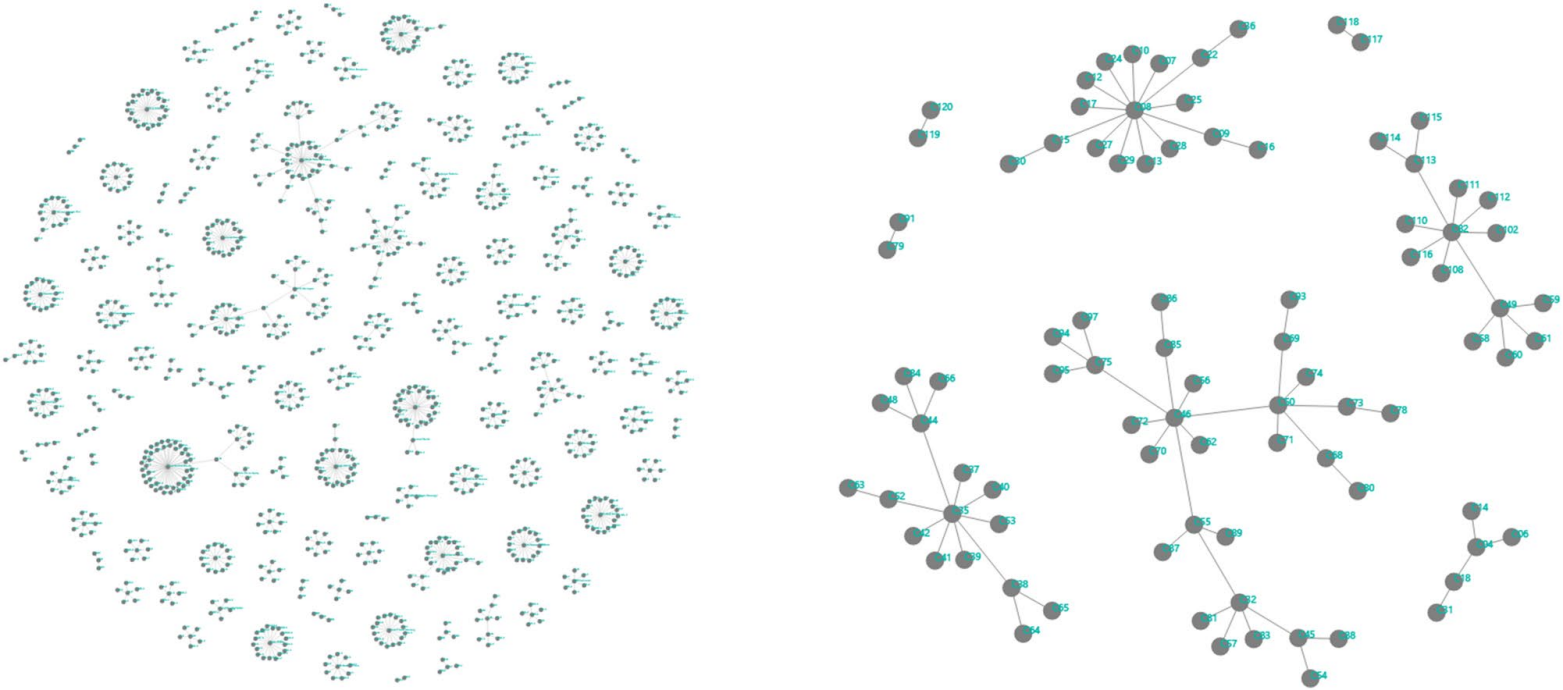
Networks of secondary infections based on local transmissions in Jakarta–Depok (left) and Batam (right) regions.

We use a sequential Bayesian method based on the discrete Susceptible-Infectious-Recovered (SIR) model to estimate the basic reproduction number $\mathscr {R}_0$^6^. Time series of the active cases at day *n* is assumed to follow the model $I_{n+1}=\left( \gamma \left( \mathscr {R}_t-1\right) +1\right) I_n$, where $\mathscr {R}_t$ is the instantaneous reproduction number and $1/\gamma$ is the infectious time taken to be 9 days herein^7^. Assuming a Poisson distribution for the arrival of active cases and using Bayes’ theorem, the posterior probability of $\mathscr {R}_t$ given a confirmed case $I_{n+1}$ is computed through

$$\begin{aligned} P\left( \left( \gamma \left( \mathscr {R}_t-1\right) +1\right) I_n|I_{n+1}\right) \propto \prod _{m=1}^nP\left( I_{m+1}|\left( \gamma \left( \mathscr {R}_t-1\right) +1\right) I_m\right) . \end{aligned}$$

**Figure 2:**
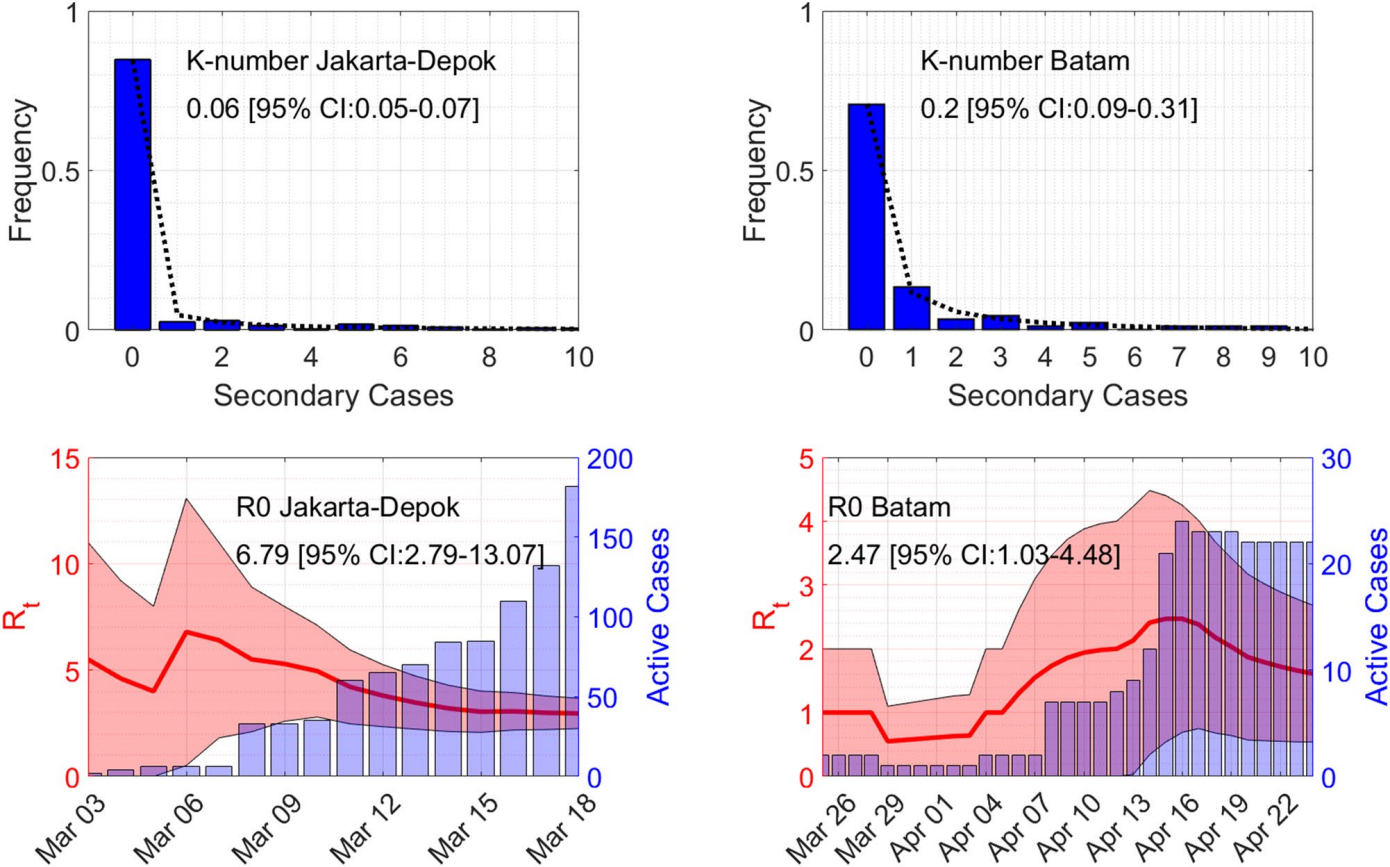
Estimates of the overdispersion parameter $\mathscr {K}$ and the basic reproduction number $\mathscr {R}_0$. Lines in the top figures show maximum-likelihood fits for the negative binomial distribution.

The formula comes from the assumption that the prior distribution of $\mathscr {R}_t$ at day *n* is taken from the posterior of day $(n-1)$ with a uniform prior at day 1. Estimated $\mathscr {R}_0$ is then the maximum of $\mathscr {R}_t$, see Fig. 2.

The parameter $\mathscr {K}$ is then defined as the overdispersion parameter in the negative binomial distribution^8^, which can be found by fitting the distribution to the secondary cases data. The overdispersion parameter describes the observation that variation is higher than would be expected. Small $\mathscr {K}$ (typically less than one) means high individual-level variation in the distribution of the number of secondary infections, and vice verse. The number of secondary cases by one person *x* can be expressed in terms of the overdispersion parameter $\mathscr {K}$ and the probability *p* as

$$\begin{aligned} P(x|\mathscr {K},p) = \frac{\Gamma (x+\mathscr {K})}{x!\Gamma (\mathscr {K})}(1-p)^\mathscr {K}p^x. \end{aligned}$$

We use a MATLAB^®^ function *nbinfit* to estimate the overdispersion parameter $\mathscr {K}$. The function uses derivative-free method to find the maximum of the likelihood function.

## Results

**Table 1:** Secondary cases data of the two regions based on local transmissions.

Jakarta–Depok		Batam	
Secondary cases	Frequency	Secondary cases	Frequency
0	1017	0	63
1	31	1	12
2	36	2	3
3,6	17	3	4
4,13	4	4.7–9.13	1
5	22	5	2
7	11		
8,11,12	3		
9	8		
10	7		
14,17,20,21,26,27,36,43	1		
15,16,19,23	2		
Total	1199	Total	89

Based on 1288 confirmed cases in March and April 2020 in the regions Jakarta–Depok (population 12.6 millions) and Batam (population 1.4 millions), we find a high degree of individual-level variation in the transmission. Early in the outbreak, superspreaders transmit the majority of coronavirus cases, as can be seen from Fig.  1. Defining a superspreader as an individual who infects at least eight other individuals, there were 44 clusters in Jakarta–Depok region (2–31 March, 2020) and 3 clusters in Batam city (19 March–7 April 2020), as can be seen from Table 1.

We estimated the reproduction number $\mathscr {R}_0$ to be at 6.79 and 2.47, while the overdispersion parameter $\mathscr {K}$ of a negative-binomial distribution is obtained at 0.06 and 0.2 for Jakarta–Depok and Batam, respectively (see Fig. 2), suggesting that a small number of infected individuals are responsible for large amounts of the disease transmission. Our finding is consistent with other researcher has found in China^9^. Indeed, between 10–15% of all infections were responsible for 80% of onward transmission events.

## Discussion

The fact that Indonesia has a moderate number of positive cases, compared to other countries with roughly the same population size such as Russia, USA, and Brazil, can be caused by three reasons: a low number of tests, the success of Large-Scale Social Restrictions, and overdispersion of the COVID-19 transmission. Our result clearly indicates that the transmission is overdispersed, even though it does not exclude the other possibilities. Therefore, close-interaction activities such as traditional markets, religious gathering, and wedding parties need to be adapted if not restricted, as they can become transmission hot spots. Effective response factors to reduce the transmission include aggressive implementation of non-pharmaceutical interventions such as rapid identification and isolation of cases. Furthermore, timeliness is critical to prevent or limit their extent since delay of diagnosis is the most common cause of superspreading clusters^10^.

## Supplementary Information

Supplementary Information 1.

## Data Availability

Secondary cases data (both in Excel and Power BI) and a MATLAB^®^ code to produce Figs.  1 and 2 are available at: https://github.com/agusisma/covidindonesia.
